# AMPK is associated with the beneficial effects of antidiabetic agents on cardiovascular diseases

**DOI:** 10.1042/BSR20181995

**Published:** 2019-02-15

**Authors:** Qingguo Lu, Xuan Li, Jia Liu, Xiaodong Sun, Thomas Rousselle, Di Ren, Nanwei Tong, Ji Li

**Affiliations:** 1 Department of Endocrinology and Metabolism, West China Hospital of Sichuan University, 610041 Chengdu, China; 2 Department of Physiology and Biophysics, Mississippi Center for Heart Research, University of Mississippi Medical Center, 39216 Jackson, MS, U.S.A.; 3 Department of Geriatrics, The First Hospital of Jilin University, 130021 Changchun, China; 4 Department of Endocrinology, Affiliated Hospital of Weifang Medical University, 261000 Weifang, China

**Keywords:** AMPK, coronary artery disease, Diabetes

## Abstract

Diabetics have higher morbidity and mortality in cardiovascular disease (CVD). A variety of antidiabetic agents are available for clinical choice. Cardiovascular (CV) safety assessment of these agents is crucial in addition to hypoglycemic effect before clinical prescription. Adenosine 5′-monophosphate-activated protein kinase (AMPK) is an important cell energy sensor, which plays an important role in regulating myocardial energy metabolism, reducing ischemia and ischemia/reperfusion (I/R) injury, improving heart failure (HF) and ventricular remodeling, ameliorating vascular endothelial dysfunction, antichronic inflammation, anti-apoptosis, and regulating autophagy. In this review, we summarized the effects of antidiabetic agents to CVD according to basic and clinical research evidence and put emphasis on whether these agents can play roles in CV system through AMPK-dependent signaling pathways. Metformin has displayed definite CV benefits related to AMPK. Sodium-glucose cotransporter 2 inhibitors also demonstrate sufficient clinical evidence for CV protection, but the mechanisms need further exploration. Glucagon-likepeptide1 analogs, dipeptidyl peptidase-4 inhibitors, α-glucosidase inhibitors and thiazolidinediones also show some AMPK-dependent CV benefits. Sulfonylureas and meglitinides may be unfavorable to CV system. AMPK is becoming a promising target for the treatment of diabetes, metabolic syndrome and CVD. But there are still some questions to be answered.

## Introduction

The prevalence of diabetes has been growing rapidly over the past 20 years. Number of diabetic patients of 20–79 years old worldwide is expected to increase to 439 million by 2030 [1]. Diabetes is considered as a significant risk factor for cardiovascular disease (CVD), the primary cause of mortality worldwide [2,3]. In 2012, about 1.5 million people died of diabetes in the world, of which about 80% were associated with myocardial infarction (MI) or stroke [4].

Diabetes treatment aims to control multiple risk factors, such as hyperglycemia, hyperlipidemia, and hypertension, etc., in order to decrease the incidence of CVD and other complications. A variety of antidiabetic agents are available clinically, for example: sulfonylureas and meglitinides, biguanides, α-glucosidase inhibitors (AGIs), thiazolidinediones (TZDs), glucagon-like peptide1 (GLP-1) analogs, dipeptidyl peptidase-4 (DPP-4) inhibitors, sodium-glucose cotransporter 2 (SGLT2) inhibitors, insulin and insulin analogs, etc. Previously, cardiovascular (CV) safety was rarely assessed in large clinical trials before antidiabetic agents were approved for market, and the majority of data obtained originally from postmarketing clinical observations. In 2007, however, an amazing result of a meta-analysis from 42 randomized trials treated with rosiglitazone, one of TZDs, was reported by Nissen and Wolski [5], which showed a possible increased risk of MI and death from CVD when comparing to the control group. This result sparked long-standing debate, although no excess CVD risk was reported in the following studies [6]. Consequently, a guidance about requiring the evidence of CV safety of novel antidiabetic agents by the pharmaceutical industry before approval was released by the US Food and Drug Administration (US FDA) [7].

Diabetic CVD is caused by many pathophysiological processes, such as macroangiopathy, microangiopathy, metabolic abnormalities, chronic inflammation, and fibrosis [8,9]. Pathogenesis and protective molecular mechanisms of diabetic CVD have been the focus of research in recent years. Adenosine 5′-monophosphate-activated protein kinase (AMPK) is an important serine and threonine protein kinase with the structure of three subunits (α, β, γ), which plays crucial roles in cell energy metabolism [10]. Increasing the ratio between intracellular adenosine monophosphate (AMP) and adenosine triphosphate (ATP), such as during strenuous exercise, hypoxia or nutritional deficiency, could phosphorylate a threonine, the 172th amino acid of the α subunit, thereby activating AMPK [11]. In addition, liver kinase B1 (LKB1), calmodulin-dependent protein kinase kinase β (CaMKKβ) and AMPK kinase (AMPKK) can be employed as upstream molecules. After activation, AMPK shuts down pathways of ATP-consuming and switches on catabolic pathways of ATP-producing through downstream signaling and target molecules [12,13], regulates lipid and protein metabolism, fatty acid oxidation, glucose uptake, gluconeogenesis, and autophagy [12–15], etc. AMPK also plays an important role in reducing oxidative stress, regulating autophagy, and anti-apoptosis of cardiomyocytes [16,17].

Our research team and others have reported that AMPK played cardioprotective roles during ischemia by increasing glucose uptake and glucose transporter 4 (GLUT4) translocation [18], decreasing apoptosis, improving postischemic recovery and limiting MI [7,19]. Furthermore, our studies have also suggested that activated protein C could activate AMPK and protect the heart from ischemia/reperfusion (I/R) injury [20], and inhibit inflammatory responses during hypoxia/reoxygenation (H/R) by modulating a JNK-mediated nuclear factor κB (NF-κB) pathway [21].

Antidiabetic agents may affect the CV system through many molecular signaling pathways. In this review, we intend to summarize the literature and discuss whether commonly used antidiabetic agents can affect CVD through AMPK-related signaling and molecular pathways.

## Sulfonylureas and meglitinides

Sulfonylureas act by binding to sulphonylurea receptor 1 (SUR1) of pancreatic β cells, and close the ATP-sensitive potassium channels (KATP), causing an augment of intracellular K^+^, triggering of membrane depolarization, opening of voltage-dependent Ca^2+^ channels, increasing intracellular Ca^2+^ influx, then inducing insulin secretion [22]. Glibenclamide, glipizide, gliclazide, and glimepiride are commonly used in clinical practice. Meglitinides, including repaglinide, nateglinide and mitiglinide, display a similar hypoglycemic mechanism (binding to SUR2) as sulfonylureas.

Concern exists regarding the CV safety of sulfonylureas [23]. In the UK Prospective Diabetes Study (UKPDS), CV mortality was similar in patients of chlorpropamide group and insulin group [24]. Glibenclamide has been associated with acute MI and mortality [25], as well as being associated with blocking the protective effects of postconditioning [26]. By contrast, gliclazide and repaglinide appear to be related a lower risk than other sulfonylureas [25, 27]. Multiple research has suggested that cardiotoxicity of sulfonylureas is associated to the closure of specific KATP channels expressed in the heart [28], which could worsen the myocardial injury. Some newer sulfonylureas may not inhibit myocardial protection. For example, gliclazide and glimepiride appear not to prevent the protective effect of ischemic preconditioning in animals [29] and humans [30].

Very little research can be retrieved on the relationship between sulfonylureas and AMPK nor meglitinides and AMPK. Glibenclamide induced a dose-dependent increase of the AMP/ATP ratio by inhibiting complexes I, II, III [31], resulting in an increased AMPK phosphorylation in H9C2 cells. However, it profoundly changes cell metabolism in cardiomyocytes by impairing mitochondrial structure and function and induces irreversible damage beyond the benefits of AMPK activation. This may further explain the risk of CV events related to this drug. However, gliclazide can increase CaMKKβ and the phosphorylated AMPK levels in vascular smooth muscle cell (VSMC) and suppress platelet-derived growth factor (PDGF)-induced VSMC proliferation by the rising of intracellular Ca^2+^ concentration [32], which is beneficial for reducing CVD.

## Biguanides

Biguanides were used for treatment of diabetes in humans in the 1920s [33] with several derivatives such as metformin, phenformin and buformin. Phenformin was withdrawn from the market in 1978 because of a rare but life-threatening side effect of lactic acidosis. Metformin is the most widely prescribed antidiabetic agent in individuals with type 2 diabetes (T2DM), which is the first-line oral therapy recommended by almost all guidelines, such as American Diabetes Association (ADA) [34], European Association of the Study of Diabetes (EASD) [35], and National Institute for Health and Care Excellence (NICE) [36], etc.

UKPDS [24] suggested that metformin reduced diabetes-related death by 42% and all-cause mortality by 36%. Similar results reported later from many clinical studies have shown CV protection and mortality reduction exerted by metformin appearing not to be dependent on its hypoglycemic effects [37–39]. Although the main antidiabetic effect of metformin was known as reducing hepatic glucose output and an increasing insulin-dependent peripheral glucose utilization [40,41], mainly by inhibiting gluconeogenesis [42], its molecular mechanism remained unclear until it was reported that it could activate AMPK in isolated hepatocytes [43]. Metformin inhibits complex I of the respiratory chain of the cell resulting in a decrease of the intracellular ATP concentration and an increase of the AMP/ATP ratio for the activation of AMPK [44–46], which is required for the CV protective effects of metformin [44,47]. Interestingly, there are also studies demonstrating that AMPK can be activated by metformin without changes in the AMP/ATP ratio [48,49] and metformin can also exert its beneficial metabolic effects on cardiomyocytes in an AMPK-independent manner [50].

Many studies suggest the pleiotropic effects of metformin mediated by activation of AMPK. We can summarize the effects of metformin on CVD through the AMPK pathways from the following aspects.

### Energy metabolism of cardiomyocyte

Impaired energy metabolism exists in many kinds of heart disease. After activation by metformin, AMPK can phosphorylate acetyl-coenzyme A carboxylase (ACC) and inhibit its function, which reduces the production of malonyl-CoA and the inhibitory effect of AMPK on carnitine palmitoyl transferase 1 (CPT1), promoting the oxidation of fatty acids [51]. In addition, activation of AMPK by metformin increases glucose uptake by inducing GLUT4 recruitment to the plasma membrane [52,53], prevents GLUT4 endocytosis and increases the residence time of GLUT4 in the plasma membrane thus increasing glucose transport and catabolism [54].

### Vascular endothelium and oxidative stress

The dysfunction of endothelial cells plays a crucial role in the occurrence and development of CVD. Metformin exerts an inhibitory effect to mitochondrial reactive oxygen species (ROS) production by selectively blocking the reverse electron flow through complex I of respiratory chain [55]. Multiple studies indicated that activated AMPK is beneficial to endothelial function by suppressing oxidative stress in endothelial cells [56,57].

Endothelial nitric oxide synthase (eNOS) has a protective function in the CV system, which is attributed to NO production regulating the vascular tone. Administration of metformin *in vivo* increases AMPK phosphorylation in the aorta of mice, resulting in increased NO synthesis, and bioavailability [58]. Metformin can also increase mitochondria-derived peroxonitrite ONOO^−^ to activate AMPK in c-Src/PI3K (phosphatidylinositol-3-kinases)-dependent manners in cultured bovine aortic endothelial cells [59]. A further study has demonstrated that AMPK activation by metformin increases the association between heat-shock protein 90 (Hsp90) and eNOS, which reduces eNOS-derived O^2−^ [60]. In addition to antioxidant stress, metformin also regulates endothelial cell energy metabolism. For example, AMPK activation by metformin increases fatty acid oxidation, which can alleviate endothelial lipotoxicity and improve endothelial function [61].

AMPK is considered as an important target for endothelial dysfunction and atherosclerosis. As an AMPK activator, metformin has great potential for promoting endothelial function to resist atherosclerosis [62]. Metformin’s CV beneficial effects of atherosclerosis prevention are mediated in part through its ability of inhibiting the oxidative stress-mediated accumulation of cholesterol via AMPK-SREBP2 (sterol regulatory element-binding protein 2)-LDLR (low-density lipoprotein receptor) axis in vascular cells [63].

### Heart failure and ventricular remodeling

Diabetes has a higher risk of developing heart failure (HF). Diabetic cardiomyopathy [64] is a common cause of HF in diabetics. It is characterized with reduced cardiomyocyte contractile function and apoptosis, mitochondrial pathology and dysfunction, and myocardial interstitial fibrosis [65].

Previously, metformin is considered contraindicated in patients with HF due to increase the risk of lactic acidosis. However, growing evidence indicates that this contraindication could be revised [66–68]. Accordingly, FDA removed the HF contraindication on the drug label for metformin in 2006, although congestive HF remains in the label’s warning section [69].

It has also been demonstrated that metformin has multiple beneficial AMPK-mediated effects in HF [70,71]. Several animal studies showed that metformin could delay the process of cardiac remodeling and the development of HF by a different pathway of AMPK activation [72]. Gundewar et al. [73] have carried out some experiments that metformin could significantly improve left ventricular (LV) function and survival by AMPK and its downstream mediators activation, peroxisome proliferator-activated receptor γ coactivator 1-α (PGC-1α) and eNOS in a murine model of HF. Chronic administration of metformin to a dog model of cardiac pacing-induced HF attenuated the hemodynamic and structural changes by AMPK activation [74]. Moreover, chronic treatment with a low dose of metformin (100 mg/kg) exerts significant cardioprotection effect against HF of rat by activating the AMPK/eNOS pathway, as well as reducing circulating and myocardial levels of insulin, transforming growth factor beta 1 (TGF-β1), basic fibroblast growth factor (bFGF), and tumor necrosis factor α (TNFα) [75].

### Myocardial ischemia and I/R injury

It has been tested that metformin and activated AMPK can play essential roles in the protection of myocardial ischemia and I/R injury by maintenance of the energy supply, and anti-oxidative stress [76]. Metformin (5 mM) in H9C2 cardiomyoblasts attenuated high glucose and H/R-induced cell injury, mitochondrial dysfunction, ROS over generation and inflammatory response through an AMPK/JNK-dependent signaling pathway [77]. A meta-analysis with 38 animals treated with metformin and 50 controls showed that the average infarct area at risk was reduced from 47.8 in the ischemia control group to 29.4 in the metformin group [78].In the study of isolated rat hearts, during the first 15 min of reperfusion metformin reduced infarct area with approximately 40–50% [79] by increased AMPK phosphorylation. Yin et al. [69] have also shown the reduction of the infarct size by metformin through AMPK phosphorylation in rats independent of systemic glucose levels. Metformin can also prevent acute death of cells in cardiac allografts by mainly suppressing intrinsic apoptosis due to I/R injury incurred from the transplantation procedure by AMPK activation [80].

### Chronic myocardium inflammation

Recent studies have indicated that metformin has a direct anti-inflammatory action by inhibition of NF-κB via AMPK-dependent and independent pathways [81]. AMPK activation by short-term administration of metformin and the subsequent suppression of Toll-like receptor 4 (TLR4) expression and activity can suppress inflammatory responses and protect the infarcted heart [82]. However, Soraya et al. found that low dose pre-treatment of metformin chronically could suppress TLR4 signaling, inhibit the release of inflammatory mediators, and reverse LV contractile dysfunction in the setting of MI in an AMPK-independent manner [83].

### Apoptosis

Cardiomyocyte apoptosis is common in CVD and diabetic cardiomyopathy. Experimental evidence suggested that metformin reduced the production of pro-apoptotic proteins, increased the anti-apoptotic proteins, and attenuated the percentage of apoptotic cardiomyocytes [84]. High-fat-induced cardiomyocyte apoptosis was partly blunted by metformin associated with increased AMPK phosphorylation [85]. Doxorubicin, a chemotherapy medication used to treat some cancer, can cause cardiotoxicity, and cardiomyocyte apoptosis. Metformin protected adult mouse cardiomyocytes (HL-1 cells) from doxorubicin-induced oxidative stress and apoptosis by modulating the expression of the adiponectin system via AMPK-mediated signaling [86]. Another research suggested that cardioprotective effects of metformin are mediated by AMPK activation, protein kinase A (PKA), Src, and platelet-derived growth factor receptor (PDGFR) [87].

### Autophagy

Autophagy is a self-degradative process that is important for balancing sources of energy at critical times in development and in response to nutrient stress [88]. Declined AMPK activity and the following reduction in cardiac autophagy are central to the development of diabetic cardiomyopathy. Metformin significantly improved mitochondrial respiration and ATP synthesis of cardiomyocytes by an underlying mechanism requiring the AMPK activation and its downstream mediators eNOS and PGC-1α [73]. Xie et al. [72] found that AMPK activated by metformin stimulates autophagy activity in cardiomyocytes by modulating Beclin1 and the tuberous sclerosis complex (TSC) mammalian target of rapamycin (mTOR) pathway in OVE26 mice. Meanwhile, a study in a murine model demonstrated that activation of Pink1-AMPK signaling by metformin rescued against phosphatase and tensin homolog (PTEN) deletion-induced changes in myocardial geometry, function, and autophagy [89].

**Figure 1:**
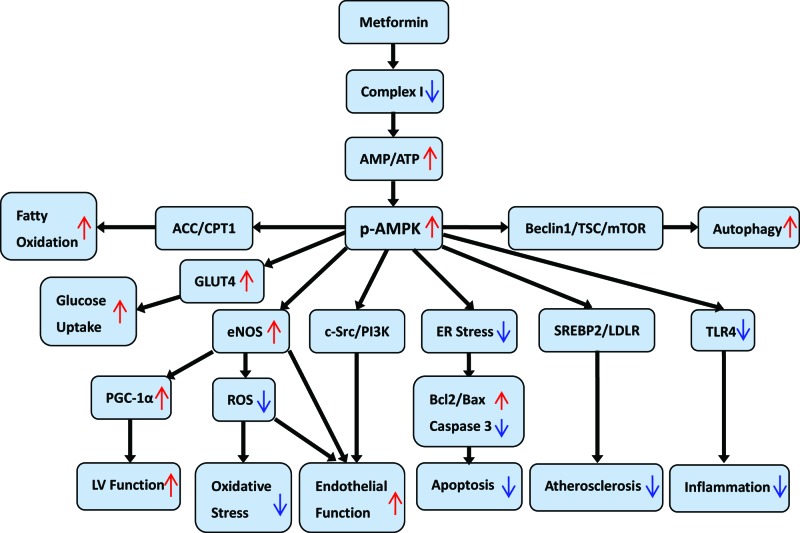
Potential downstream molecular signaling pathways of AMPK activated by metformin on cardioprotection Abbreviations: ER, endoplasmic reticulum; p-AMPK, phosphorylated AMPK.

Downstream molecular signaling pathways of AMPK activated by metfromin for the protection of CV system are shown in Figure 1.

## α-Glucosidase inhibitors

AGIs are a type of widely used hypoglycemic agents with the mechanism of delaying the absorption of carbohydrates from the upper part of small intestine and producing a lowering effect to postprandial blood glucose. Acarbose, miglitol, and voglibose are involved.

Some clinical and basic studies have provided evidence of CV protection of AGIs. Acarbose reportedly reduced the morbidity of hypertension, CV events [90], and silent MI [91]. It also slowed progression of intima-media thickening of individuals with impaired glucose tolerance (IGT), improved carotid plaque echogenicity in patients with acute coronary syndrome (ACS) [92,93], and reduces the risk of MI in T2DM patients [94]. Acarbose could also stabilize carotid plaque within 1 month of therapy in patients with ACS and T2DM [93]. In the reports of basic research, acarbose reduced MI size in animals by opening mitochondrial KATP channels [95]. Some reports have suggested that the absorbed miglitol suppressed neointimal thickening of the arterial wall in animals [96,97]. Voglibose significantly decreased infarct size of nondiabetic rabbits during 30 min of ischemia and 48 h of reperfusion condition [98] by up-regulating GLP-1 levels and activating the GLP-1 receptors, with downstream activation of Akt, eNOS, and the mitochondrial KATP channels.

Research of AGIs on CVD through AMPK pathway is rare. Acarbose could improve vascular inflammation by enhancing NO expression to suppress cell cycle progression and inhibiting VSMC proliferation through AMPK activation and Ras inhibition, thus preventing or slowing the development of atherosclerosis [99]. Miglitol inhibits endothelial cell injury and protects against DNA damage under intensive oxidative stress, which may be involved in the activation of AMPK in endothelial cells, with the result of increasing NO production and reduced intercellular cell adhesion molecule-1 (ICAM-1), and vascular cell adhesion molecule-1 (VCAM-1) mRNA expression [100]. Mulberry 1-deoxynojirimycin (DNJ) can be considered as an AGI analog with the effect of inhibiting α-glucosidase in the small intestine [101,102]. Chan’s group has indicated the mechanisms by which mulberry leaf DNJ effectively inhibit proliferation and migration of VSMCs, including AMPK/RhoB activation and down-regulation of FAK *in vitro* study [103].

## Thiazolidinediones

TZDs increase insulin sensitivity through binding of the so-called peroxisome proliferator-activated receptor γ (PPAR-γ) to activate downstream genes that are involved in glucose and fatty acid metabolism. Of the members in this family, troglitazone has been withdrawed from the market, but rosiglitazone and pioglitazone remain in use.

In clinical studies, the effects of rosiglitazone and pioglitazone on CVD display diversity. Previous meta-analysis suggested that rosiglitazone use is associated with the risk of MI and death from CVD, and also increase risk of fluid retention which may exacerbate HF [5,104]. In a retrospective, observational trial, rosiglitazone was related with an increased risk of stroke, HF, and a composite outcomes of acute myocardial infarction (AMI) [105]. However, the prospective Rosiglitazone Evaluated for Cardiac Outcomes and Regulation of Glycemia in Diabetes (RECORD) trial found that there is no adverse effect of rosiglitazone on the composite outcome of CV death or hospitalization but report an increased risk of hospitalization for congestive heart failure (CHF) in patients using rosiglitazone versus active comparator [6]. Rosiglitazone has beneficial CV effects of nondiabetics in basic research [106]. Other clinical studies [107] and a meta-analysis [108] have demonstrated that pioglitazone reduces CV complications in individuals of T2DM. Despite the FDA’s release of rosiglitazone use and prescription restrictions in 2013 due to later clinical trial evidence [109,110], patients with HF are still very cautious to use.

TZDs are ligands for the nuclear hormone receptor family member PPAR-γ [111]. Both rosiglitazone and pioglitazone have been proved to activate AMPK in intact cells [112,113] by stimulating the release and expression of circulating adiponectin from adipose tissue [114–116], or indirectly by enhancing the cellular AMP/ATP ratio, possibly via a similar mechanism with biguanides [117]. Our group conducted a series of studies on the role of TZDs and AMPK in the heart. The result demonstrated that by using rosiglitazone acutely under I/R stress, MI is decreased and postischemic cardiac function is improved by modulating AMPK, Akt, and JNK signaling mechanisms in the nondiabetic mouse heart [118].

Many studies have suggested that TZDs can attenuate myocardial hypertrophy mediated by AMPK. It has been shown that adiponectin stimulates the phosphorylation of AMPK and suppresses agonist-stimulated extracellular regulated protein kinases (ERK1/2) activation and hypertrophic response in cultured cardiomyocytes through activating AMPK signaling [119]. Antihypertrophic effect of pioglitazone is attributed to reduced ERK1/2 activation that is involved in the adiponectin-AMPK regulatory axis [120]. Administration of pioglitazone with long term delayed the development of LV hypertrophy and fibrosis as well as inhibited phosphorylation of mTOR and p70S6 kinase in the heart, which are likely attributable, at least in part, not only to the AMPK activation through stimulation of adiponectin secretion but also to the Akt signaling inhibition in the heart [121].

TZDs also plays an essential role in adjusting energy metabolism. Troglitazone could significantly increase glucose uptake and activated both AMPK and eNOS signaling in isolated papillary muscles [122]. Adiponectin stimulated by TZDs can bind to adiponectin receptor 1 (AdipoR1) and activate AMPK/ACC/CPT-1 pathway to enhance fatty acid β-oxidation in the heart, a pathway that is also regulated by PGC-1α and PPARα [123]. However, rosiglitazone does not always increase glucose and fatty acid metabolism in adiponectin/AMPK pathway. An interesting research showed that activation of PPAR-γ in the late-gestation sheep fetus, rosiglitazone may decrease cardiac metabolism (glucose uptake, fatty acid β-oxidation) and cardiomyocyte size by down-regulating AdipoR1, phospho-AMPK, phospho-ACC, and PGC-1α [124].

## GLP-1 analogs

GLP-1 is a type of incretin hormone that is secreted from L-cells of the small intestine in a glucose-dependent manner to stimulate insulin secretion, increase pancreatic β cell mass, and inhibits glucagon secretion and gastric emptying, thus reducing postprandial glycemia [125,126]. Due to rapid degradation by DPP-4, the half-life of endogenous GLP-1 is very short. Longer half-life synthetic analogs have been developed for clinical use as a new class of antidiabetic agents, such as: exenatide, liraglutide, lixisenatide, albiglutide, dulaglutide, and semaglutide, etc.

Several large clinical studies have revealed that GLP-1 analogs can reduce the risk of major adverse cardiovascular events (MACE), nonfatal MI, and CV death, etc., in T2DM [127–130]. GLP-1 analogs play CV protective roles mediated by GLP-1 receptor (GLP1R) in CV tissues, among which, activation of AMPK signaling pathway is still crucial. Considerable evidences demonstrate that GLP-1 protects the isolated mouse heart against I/R injury by AMPK pathway [131]. GLP-1 analogs have also been shown to exert direct cardioprotective effects of MI in murine models [132]. Liraglutide could increase AMPK phosphorylation in the hearts of obese mice with the similar effect to metformin [73]. A short-term treatment with a weight-neutral dose of liraglutide can reverse the molecular pathophysiology of obesity-induced heart disease in mice through a variety of putative mechanisms with a central role for AMPK [133]. GLP-1 analogs can also display the effect in balancing energy metabolism and maintaining heart function of diabetic models. Guo’s [134] study showed that exenatide treatment increased the level of phosphorylation of AMPK and the mRNA expression of GLUT4 in the diabetic heart of rats. The increased adiponectin may partially explain these change, which might contribute to the ameliorated heart function. After being treated with exenatide, the adiponectin and high-molecular-weight-adiponectin and the APPL1-AMPK-PPARα axis were increased, the NF-κB and the apoptosis were decreased, and the cardiac function of the diabetic rats was improved [135].

We have discussed the essentiality of autophagy for cell survival above. Liraglutide relieved myocardial damage by enhancing autophagy via AMPK-mTOR signaling pathway in Zucker diabetic fatty rat [136]. In adult rat cardiomyocytes, GLP-1 activates AMPK, then inhibits the hyperglycemia-induced NADPH oxidase 2 (NOX2) activation by limiting protein kinase C (PKC) phosphorylation and p47phox translocation to the caveolae; thereby, preventing glucotoxicity [137]. Abbas and Kabil [138] demonstrated that liraglutide treatment had been shown to relieve doxorubicin-induced cardiotoxicity, may be attributed to the antioxidant and anti-inflammatory effects as well as anti-apoptotic effects through the AMPK/Akt/GSK-3β (glycogen synthase kinase 3β) signaling pathway.

Meanwhile, administration of liraglutide protected against myocardial steatosis and oxidative stress by activation of the AMPK-Sirt1 (silent mating type information regulation 2 homolog) pathway [139] at least in part. GLP-1 analogs can also improve endothelial dysfunction. Liraglutide plays an anti-inflammatory role to primary human aortic endothelial cells (HAECs) by causing a subsequent increase in intracellular calcium, CaMKKβ activity and AMPK activation [140]. Exenatide significantly improves coronary artery endothelial function of individuals with newly diagnosed T2DM. The improvement effect may be mediated by activation of the AMPK/PI3K-Akt/eNOS pathway via a GLP-1R/cAMP-dependent mechanism [141].

## DPP-4 inhibitors

DPP-4 inhibitors are a group of agents for treating T2DM [126]. They prevent the deactivation of the two endogenous incretine hormones, GLP-1 and glucose-dependent-insulinotropic-peptide (GIP), thus causing the accumulation of these hormones [142] to make these hormones play the role of antidiabetes. Sitagliptin, saxagliptin, vildagliptin, alogliptin, linagliptin, and trelagliptin are approved on market as DPP-4 inhibitors family members. Several large clinical studies have shown that DPP-4 inhibitors do not increase the risk of CVD in type 2 diabetics compared with placebos [143–145].

It has been reported that DPP-4 inhibitors can limit infarct size in the nondiabetic mice [146] and isolated rat hearts [147]. Sitagliptin has been reported to play a protective role in CVD and atherosclerosis [148,149]. Sitagliptin can decrease the atherosclerotic lesion area by activating AMPK-mediated Akt signaling pathway in ApoE^−/−^ mice while attenuating the phosphorylation of p38 and ERK1/2 and mitogen-activated protein kinase (MAPK), therefore, inhibiting inflammatory responses in the aorta, such as the release of monocyte chemoattractant protein 1 (MCP-1) and interleukin 6 (IL-6), and the expression of the VCAM-1 and serum P-selectin [150]. One study indicated that sitagliptin inhibits endothelin-1 (ET-1) expression in the aortic endothelium by suppressing the NF-κB/IκBα system through the activation of the AMPK pathway in diabetic rats, which demonstrated some of the vasoprotective properties of DPP-4 inhibitors *in vivo* [151]. In other studies, sitagliptin prevented hyperglycemia induced apoptosis via activation of AMPK in HUVECs and also attenuated myocardial apoptosis by activating LKB-1/AMPK/Akt signaling pathway and suppressing the activity of GSK-3β and p38α/MAPK in diabetic cardiomyopathy of rat [152].

However, Lenski’s group found that [153] sitagliptin treatment reduced the increased phosphorylation of AMPK and ACC in db/db^−/−^mice, then reduced membrane translocation of GLUT4 in cardiomyocytes, thus prevented the metabolic alteration associated with the diabetes-obesity syndrome via AMPK and its downstream molecule in the myocardium.

## SGLT2 inhibitors

SGLT2 inhibitors selectively inhibit SGLT2 of the renal proximal tubule, with a consequent decrease in renal tubular thresholds for glycosuria and increase in urinary excretion of glucose, reducing blood glucose independently of insulin. Canagliflozin, dapagliflozin, empagliflozin, and ertugliflozin of this family have been approved by FDA.

As the request of FDA, some clinical trials for CV risk assessments were conducted before market. EMPA REG OUTCOME (Empagliflozin, Cardiovascular Outcomes, and Mortality in type 2 diabetes) demonstrated that empagliflozin exerts a 38% risk reduction in death from CV causes, 32% risk reduction death from any cause and 35% reduction on risk of hospitalization for HF [154]. CVD-REAL study has confirmed that the positive effects on HF of SGLT2 inhibitors can be considered a class effect, not only by empagliflozin [155]. CANVAS study for evaluating canagliflozin also showed similar CV protective effect [156]. FDA has approved a new indication for empagliflozin to reduce the risk of CVD death in adult patients with T2DM and CVD in December, 2016 [157].

After clinical analysis, the protective effect of SGLT2 inhibitors on heart may be related to lowering blood pressure, weight loss, decreasing serum uric acid level, osmotic diuresis, reducing volume load and hemodynamic changes, etc. Further molecular mechanism is still in the exploratory stage. At present, basic research are focusing on energy metabolism, inflammation, oxidative stress, myocardial fibrosis and electrolyte homeostasis [158]. SGLT2 inhibitors change the energy metabolism of the heart from glucose to fat [159–161] and slightly increase the ketone level [162], which is beneficial for cardiac energy supply during HF. Empagliflozin can significantly improve myocardial fibrosis in obese and diabetic mice [163,164], and also play the role of anti-oxidative stress and anti-apoptosis [165,166]. Meanwhile, empagliflozin can reduce infarct size after I/R [167], and improve diastolic function of the left ventricle in diabetic mice [168]. Dapagliflozin can delay the occurrence and progress of diabetic cardiomyopathy [169].

There are currently relatively few studies on SGLT2 inhibitors and AMPK. Canagliflozin activates AMPK human embryonic kidney (HEK-293) cells and hepatocytes by inhibiting complex I in the mitochondrial respiratory chain and increasing cellular AMP levels [170]. Clinically-relevant canagliflozin concentrations can directly inhibit endothelial pro-inflammatory chemokine/cytokine secretion by AMPK dependent and independent mechanisms without affecting early interleukin-1β (IL-1β) signaling [171]. Dapagliflozin decreases the activation of the NOD-like receptor family, pyrin domain containing 3/apoptosis-associated speck-like protein containing a CARD (NLRP3/ASC) inflammasome attenuated myocardial inflammation, fibrosis, apoptosis, and diabetic remodeling likely mediated through AMPK activation [164]. In another study, empagliflozin alleviated diabetic cardiac microvascular endothelial cell (CMEC) injury by inhibiting mitochondrial fission via the activation of AMPK-Drp1 (Dynamin-related protein 1) signaling pathways, preserved cardiac CMEC barrier function through suppressed mitochondrial ROS production and subsequently oxidative stress to inhibit CMEC senescence. So empagliflozin can be considered as a cardiac microvascular-protection agents to maintain cardiac circulatory function and structure upon hyperglycemic insult [172].

Our group is currently studying the molecular mechanisms underlying the cardioprotective effect of empagliflozin. Preliminary results show that a certain concentration of empagliflozin can enhance the contractility of isolated mice cardiomyocytes under the condition of intracellular hypoxia. At baseline, empagliflozin can phosphorylate AMPK in mice cardiomyocytes. In intracellular hypoxia state induced by sodium cyanide (NaCN), empagliflozin can prolong AMPK activation time of mice cardiomyocytes (unpublished data). The further molecular mechanisms of AMPK activation is still under exploration.

**Figure 2:**
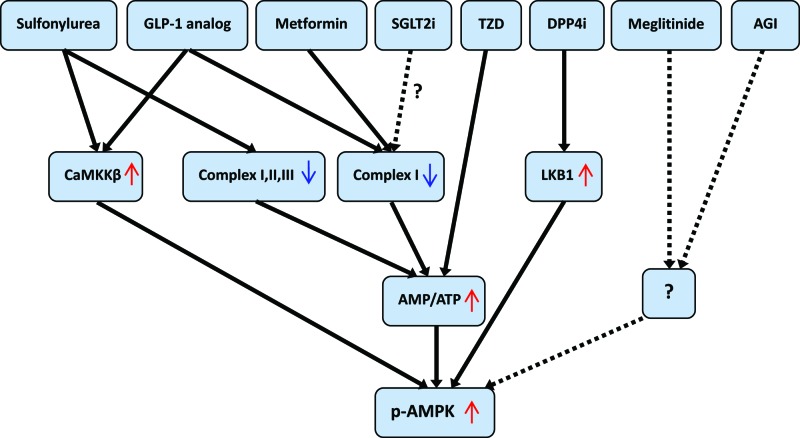
Upstream signaling pathways of AMPK activation by antidiabetic agents Abbreviations: DPP4i, DPP-4 inhibitors; p-AMPK, phosphorylated AMPK; SGLT2i, sodium-glucose cotransporter 2 inhibitors.

We summarized the upstream signal pathways of AMPK activated by antidiabetic agentsin CV system in Figure 2.

## Pespectives

We enumerate the CV safety of commonly used antidiabetic agents in clinical practice and summarize whether these drugs can affect CVD through AMPK-related signaling pathways in this review to help clinicians for selection of antidiabetic agents. The mechanism of metformin on activating AMPK is relatively clear, but it is still obscure for other antidiabetic agents. Therefore, further research is needed to find answers from the intricate AMPK signal transduction network. Given the benefits of AMPK activation for diabetes and CVD, AMPK is becoming a promising target for the treatment of diabetes, metabolic syndrome, and CVD. There are still some problems to be solved. First, AMPK has many subtypes, and the expression of each subunit is different among species and tissues, making it more difficult to translate AMPK activators from pre-clinical animal experiment to clinical trial. Second, AMPK is expressed in many organs and tissues of the whole body. Whether systemic activation caused by nonspecific AMPK agonists has adverse effects on some organs is unknown. Therefore, it is necessary to develop organ-specific AMPK agonists. Third, the AMPK signaling network is very complicated, and there are many cross-talk with other pathways. The effect of activating AMPK on other pathways also needs a lot of research to confirm. At last, what is the activation duration and degree of AMPK? It is also unknown whether excessive or prolonged activation will bring adverse effects. Therefore, there is still a long way to go on progressing AMPK from basic research to clinical application.

## Abbreviations

ACC: coenzyme A carboxylase
ACS: acute coronary syndrome
AdipoR: adiponectin receptor
AGI: α-glucosidase inhibitor
AMPK: adenosine 5′-monophosphate-activated protein kinase
CaMKK: calmodulin-dependent protein kinase kinase
CMEC: cardiac microvascular endothelial cell
CV: cardiovascular
CVD: cardiovascular disease
DNJ: deoxynojirimycin
DPP: dipeptidyl peptidase
eNOS: endothelial nitric oxide synthase
ERK: extracellular regulated protein kinase
GLP: glucagon-like peptide
GLUT: glucose transporter
GSK: glycogen synthase kinase
H/R: hypoxia/reoxygenation
I/R: ischemia/reperfusion
KATP: ATP-sensitive potassium channel
LDLR: low-density lipoprotein receptor
LKB: liver kinase B
MAPK: mitogen-activated protein kinase
mTOR: mammalian target of rapamycin
NF-κB: nuclear factor κB
PGC: peroxisome proliferator-activated receptor γ coactivator 1-α
PI3K: phosphatidylinositol-3-kinase
PPAR: peroxisome proliferator-activated receptor
ROS: reactive oxygen species
SGLT: sodium-glucose cotransporter
SREBP2: sterol regulatory element-binding protein
T2DM: type 2 diabetes
TLR: Toll-like receptor
TZD: thiazolidinedione
UKPDS: UK Prospective Diabetes Study
VCAM: vascular cell adhesion molecule
VSMC: vascular smooth muscle cell
